# Academic stress and suicidal ideation: moderating roles of coping style and resilience

**DOI:** 10.1186/s12888-022-04063-2

**Published:** 2022-08-12

**Authors:** Franca Obiageli Okechukwu, Kalu T. U. Ogba, Juliet I. Nwufo, Miracle Oluchi Ogba, Blessing Nneka Onyekachi, Chinonso I. Nwanosike, Amuche B. Onyishi

**Affiliations:** 1 Department of Home Science and Management, University of Nigeria, Nsukka, Nigeria; 2 Psychology Department, University of Nigeria, Nsukka, Nigeria; 3 Faculty of Law, Abia State University, Uturu, Umuahia, Nigeria

**Keywords:** Academic stress, Suicidal ideation, Coping, Resilience, Moderation

## Abstract

**Background:**

As a global phenomenon, suicide has generated a lot of concern. Scholars from various fields have conducted extensive research on the prevalence, causes, factors, and/or management or possible solutions to suicidal ideation. Despite the research efforts, suicidal cases worldwide still yell for more empirical attention. No doubt that some of the extant literature have specifically evidenced the causal links and factors in suicidal ideation. Yet, none had focused on the moderating roles of coping and resilience in an academic population. We therefore, examined the moderating roles of coping and resilience in the relationship between academic stress and suicidal ideation.

**Method:**

We used a cross-sectional design to sample 505 participants (329 males and 176 females) from three southern Nigerian universities. Participants who willingly indicated their participatory consent were administered a paper self-report questionnaire containing the Lakaev Academic Stress Response Scale (LASRS), Scale for Suicidal Ideation (SSI), Brief COPE (B-COPE), and Resilience Scale (RS-14). Hierarchical regression analysis was used to test the hypotheses of the study.

Academic stress (*r* = 0.17; p.001) was found to be positively associated with suicidal ideation, whereas resilience (*r* = −.22; p.001) was found to be negatively associated with suicidal ideation. Suicidal ideation had no significant correlation with adaptive coping style, but it did have a significant correlation with maladaptive coping (*r* = .15; p.001). The regression-based PROCESS macro showed that academic stress was a significant predictor of coping [Δ*R*^2^ = .03, F (1, 502) **=** 16.18, *p* = .01]. Academic stress was positively associated with suicidal ideation at low or moderate levels of adaptive coping styles. At high levels of adaptive coping styles, the association between academic stress and suicidal ideation was not significant. However, resilience negatively predicted suicidal ideation [R = .29, (R^2^ = .08), F(1, 499) = 19.94, *p* = .00] with academic stress showing a positive association with suicidal ideation at low and moderate levels of resilience, but for those with high resilience, academic stress was not associated with suicidal ideation.

In sum, suicidal ideation is heightened by increased academic stress, with greater resilience ameliorating the tendency of academic stress resulting in suicidal ideation. Also, adopting maladaptive ways of coping promotes suicidal ideation among students, with resilience and adaptive coping strategies moderating the relationship between academic stress and suicidal ideation. It is therefore recommended that educational administrators, policy makers, lecturers, teachers, and tutors incorporate courses, teachings, and sessions that foster as well as inculcate resilience and efficient coping skills in pupils and students.

## Introduction

Suicide is multifarious and a major concern for public health [58, 74]. It is a diverse, less comprehensible, life-threatening phenomenon. This is because most victims of suicide hide or conceal their intentions [11, 25, 70], and this makes it difficult (if not impossible) for people to have knowledge of or even gain access to a potential suicide victim. As a result, WHO [73] noted that one person dies by suicide every 40 seconds despite progress in national prevention strategies. Consequently, it has become the second leading cause of death among youths. Snowdon and Choi [58] observed that reports of suicide are rare in children under the age of 10, but in the developed world, the prevalence begins to increase for youths between 10 and 14 years of age and in the 15 to 24 year age group [14]. Among their Nigeria counterparts, Adewuya and Oladipo [1] observed that the prevalence is 13–29 years, while the Nigeria National Youth Policy [43] discovered that the prevalence is in the 18–35 year age group. Due to cultural and developmental differences across individuals, there are inconsistencies as to the exact age at which suicidal ideation occurs. This could account for why scholars like [1, 4, 41] noted that evidence from 32 low and middle-income countries in sub-Sahara Africa have high suicide rates among adolescents and young people in general (without reference to a particular age bracket). Uganda, Botswana, Kenya, Zambia, and Nigeria have high prevalence of suicidal ideation among young people [55]. These youths within “transitory-into-productive” age(s), are seen to be moving from tertiary institutions into the uncertain world of labor markets in the developing (and in some western) worlds. Besides, suicide seems to have had multiple underlying causes [9, 67], and therefore requires adopting multiple investigative approach. Hence, our study investigated the moderating impacts of coping and resilience on academic stress and suicidal ideation among students.

Stresses associated with completing tertiary education, as well as concerns about unemployment, poverty, destitution, economic crises, feelings of insecurity, marginalization (including biases), and economic disempowerment [8, 34, 44], are as prevalent in society as the need for adequate coping knowledge [8, 34, 44]. Failure to adequately cope greatly increases the chances of severing youths from the traditional values and moral regulations that seemed to have earlier provided moral foundation and guide, leading to thoughts of suicide. This could account for scholars’ reports (e.g. [27, 36]) that suicidal thoughts are more common among younger age groups.

Stress is no longer new to people as it has permeated every aspect of humanity. Hence, the present study would emphasize academic stress. Undergraduateship is not devoid of challenges and stressful circumstances. These circumstances are not limited to adapting to a new academic environment, academic workload, academic performance, attending to lectures, overwork, future employment [22, 49], nor social and financial stresses [19]. Whether these stressors are short-term or long-term, they have significant impact on undergraduates’ coping (either adaptive or maladaptive) capacity [19]. Productive (adaptive) coping protects students from suicide and suicidal ideation [10, 18]; whereas ineffective/dysfunctional (maladaptive) coping skills among students experiencing persistent academic stress and negative emotions trigger higher risk of suicide. The relationship between academic stress and suicidal ideation has been well documented in literature (e.g [31, 48, 66]). Generally, the role of stressful life events in suicidal ideation, attempts, and completion has been a key area of study in the epidemiology of mental disorders [35, 69]. There is a need to understand the moderating roles of some factors in the observed association [45] between academic stress and suicidal ideation in a bid to advance research knowledge on suicide, intervention, and treatment. This is an important contribution that the current study offers to the body of knowledge. Since stress has been implicated in suicide [31, 48], with no drugs for identified victims of suicidal ideation, coping is very germane.

Lambert and Lambert [38] noted that coping is a conscious effort to reduce stress, and entailing masterful ways of tolerating, reducing, or minimizing stressful events. The conceptualization and categorization of different coping styles is inconsistent in literature (cf. [57]). Notwithstanding divergent opinions on conceptions of coping, coping has colossal impacts on stress (academic not exempted) and suicidal ideation. For instance, behavioural disengagement and self-blame increase suicidal vulnerability [30], deficient coping and problem solving skills heighten suicidal ideation [59, 62], passive coping (usually fantasizing) fosters suicidal ideation [75], while ineffective coping skills and negative emotions trigger higher risk of suicide [15]. Coping skills such as active coping and positive reframing were negatively associated with suicide, whereas coping skills like self-distraction, substance abuse, behavioural disengagement, venting, and self-blame were positively associated with suicide (e.g. [39]).

Besides these direct associations, psychopathological factors, including depression [68], hopelessness [21], and psychological distress [63], have been tested as mediators between life stress and suicidal ideation, with fewer research enquiries involving resilience. Resilience is an individual’s tendency to bounce back to a previous state of normal functioning, or simply not showing negative effects after stress and adversity. Wagnild [71] noted that resilience is an ability to recover from stress. As a helpful behavioural disposition, it promotes an individual’s healthy survival and soothes the negative outcome of stress. Resilience is important as it ensures healthy social functioning, morale, and somatic health, as well as helps an individual maintain emotional stability in the midst of stress [64]. Hence, understanding resilience appears to provide homeostasis [51] and personal endurance [33]. A study [40] on the relationship between resilience and well-being associated resilience with a positive view of the self. Cleverly and Kidd [16] found youths’ perceived resilience related to less suicidal ideation, whereas higher psychological distress was associated with higher suicidal ideation. Furthermore, depression has been linked to suicidal ideation, with anxiety, mental health, resiliency, and daily stress playing important roles [32]. Again, resilience dimensions such as social resources and familial cohesion were strongly and negatively correlated with humiliation, interpersonal sensitivity, and depression in subjects with previous suicidal attempts [52].

To our knowledge, no study has combined coping and resilience as moderators of the relationship between academic stress and suicidal ideation. Rather, extant related literature have either focused on stress (not academic stress) and suicidal ideation [17, 20] or coping and suicidal ideation [10]. Although Zimmerman [76] provided useful theoretical explanations and understandings as to how some ‘promotive factors’ could interrupt the pathways to mental health difficulties among youths, we believe it is necessary to investigate as many of these promotive factors (including coping and resilience) as possible with respect to suicidal ideation. The present study might support as well as enhance, and further the theoretical explanations of Zimmerman [76]. However, it is important to note that some studies have investigated coping as a moderator in relationship of stress (but not necessarily academic stress) and suicidal ideation (e.g. [17, 20, 68]). We assume that coping styles will have moderating impact on suicidal ideation and academic stress among undergraduates, especially for those who adopt functional or adaptive coping styles, compared to those who do not. In the same vein, we equally propose that resilience will moderate the link between academic stress and suicidal ideation. When confronted with the aforementioned potential stressors, a student who is stressed but adopts dysfunctional or ineffective coping styles (blaming oneself for problems, ignoring them, or escaping through fantasizing thoughts) may likely consider suicide as an option to end the perturbation [26]. Those who use effective or functional strategies (positive reevaluation, planning, and seeking help) are less likely to consider suicide [39]. In other words, coping could either increase or decrease the effect of academic stress on suicidal ideation, whereas resilience helps them bounce back after having adaptively coped with academic stress. Therefore, we hypothesized first, that academic stress would predict suicidal ideation; second, while adaptive coping style would not predict suicidal ideation, maladaptive coping style would; third, adaptive coping style would moderate the association between academic stress and suicidal ideation such that at low or moderate levels of adaptive coping styles, academic stress would be positively associated with suicidal ideation; but at high levels of adaptive coping style, the relationship of academic stress and suicidal ideation would not be significant. Finally, resilience would negatively predict suicidal ideation [54] as well as moderate the association between academic stress and suicidal ideation, such that academic stress would show a positive association with suicidal ideation for students at low and moderate levels of resilience, but for those with high resilience, academic stress would not be associated with suicidal ideation.

In comparison with most western societies, single studies on the moderating roles of coping, resilience on academic stress and suicidal ideation in a Nigerian sample are very rare. Also, studies with Nigerian (and perhaps other) samples have rather dominated the areas of protective and risk factors for suicidal behaviour and ideation (e.g. [1, 2, 46, 53]). Our study is relevant because it advances the knowledge quest for preventive and management approaches for students and school administrators who may struggle to successfully navigate academic-related stress without deteriorating to suicidal ideation.

The understanding that suicidal ideation may decrease among undergraduates because of adaptive or functional coping skills; and that students who practice functional coping skills may suppress the negative experiences, anxiety, and psychological distress that emanate due to academic stress, is very crucial in proposing and inculcating a positive academic survival approach. This outcome could equally be transferred into other domains of students’ lives even after school. It is also essential to policymakers, educational administrators, parents, students, and society at large as no one is exempted from the scorching heat of rampant suicide among undergraduates- a generational transitory population. Therefore, the study encourages stakeholders to teach and practice adaptive coping skills as well as resilient techniques whose ripple effects not only reduce suicidal ideation but also help in healthy living.

## Method

### Participants and procedure

The study adopted a cross sectional design to sample a total of 505 undergraduates from three South-Eastern universities in Nigeria. They consisted of 329 (65.1%) males and 176 (34.9%) females who were conveniently sampled at their clustering and administered a self-report battery of measures. Out of the five federal universities in the Southeast, three universities were randomly selected using a table of random numbers. The three universities were: the University of Nigeria, Nsukka (UNN), the Alex Ekwueme Federal University, Ndufu Alike Ikwo (AE-FUNAI), and the Michael Okpara University of Agriculture. The University of Nigeria, Nsukka was founded by Nnamdi Azikiwe in 1955 and formally opened in 1960. UNN has more than nine faculties, including the faculties of Agriculture, Arts, Biological Sciences, Education, Engineering, Pharmaceutical Sciences, Physical Sciences, Social Sciences, Veterinary Medicine, a School of General Studies, etc. The Alex Ekwueme Federal University Ndufu Alike Ikwo (AE-FUNAI) is located in Ndufu, Alike Ikwo in Ebonyi State, Nigeria. It was established in 2011. Courses offered include: Agriculture, Basic Medical Sciences, Education, Engineering and Technology, Humanities, Management Sciences, Social Sciences, Biological Sciences, Environmental Sciences, College of Medicine, Physical Sciences, Law, etc. The Michael Okpara University of Agriculture is located in Umudike, Abia State, Nigeria and was established as a specialized university in 1992. Education, Veterinary Medicine, Applied Food Science and Tourism, Agricultural Economics, Rural Sociology, Extension, Animal Science & Animal Production, Physical & Applied Sciences, Natural Resources & Environmental Management, Natural Sciences, Management & Social Sciences, Engineering & Engineering Technology, Crop & Soil Sciences, and Humanities [13].

In terms of setting, these universities were similar. In that, the establishment of a university automatically transforms even the most rural of places into an urban setting. However, these universities differed in terms of courses offered and socio-economic status. At the selected federal universities, participants were met at their various hostels and lecture quadrangles. Those who indicated their participatory consent prior to the creation of rapport were administered the self-report battery of measures. Age, sex, ethnic group, marital status, and educational qualifications were assessed through the self-report battery of measures. Participants were asked to indicate by ticking in the appropriate boxes their age (in years); sex (male and female); ethnic group (Igbo, Hausa, Yoruba, and others); marital status (single and married); and educational qualification. Educational qualification was removed from the analysis because the participants were still undergraduate students. The four instruments were prepared in a questionnaire format. A brief statement of consent that sought the participant’s consent was attached to the questionnaire. Participants were expected to first read through the brief consent letter and indicate their participatory consent by ticking on the appropriate boxes. Those who declined their interest in participation in the consent letter were asked to kindly return the questionnaire. The questionnaires were administered on a one-on-one basis and retrieved upon completion [29]. In addition to the consent letter, the questionnaires were distributed to students who willingly accepted to take part in the study, with a preceding self-introduction and explanation of the objective of the study. Participants were verbally appreciated. Out of the 530 copies of the questionnaire distributed, 523 were returned (98.7% return rate), while 18 were discarded due to improper completion. To preserve the homogeneity of the sample, all participants were undergraduates, irrespective of other demographic characteristics. Our procedures met relevant ethical guidelines and legal requirements in Nigeria to warrant the ethical approval obtained on (November 21, 2019) from the Institutional Review Board, University of Nigeria, Nsukka.

### Measures

#### Lakaev academic stress response scale (LASRS [37]) 

The LASRS is a 21-item structured scale that measures students’ responses to stress in physiological, behavioural, cognitive, and affective domains. Respondents rated how much of the time they experienced symptoms on a 5-point Likert scale [37] with the anchors: None of the Time (1), A Little of the Time (2), Some of the Time (3), Most of the Time (4), and All of the Time (5). Items were summed for subscale scores, and subscales were summed for a total LASRS stress response score. Higher scores indicated a greater stress response. It has excellent psychometric properties with internal consistency ranging from .64 to .92 [37]. Our pilot testing of the scale yielded a Cronbach’s alpha of .83.

#### Scale for suicidal ideation (SSI [6])

SSI is a 19-item self-report scale designed to quantify the intensity of current conscious suicidal intent, by scaling various dimensions of self-destructive thoughts or wishes. The items assessed the extent of suicidal thoughts and their characteristics, as well as the respondent’s attitude towards them; the extent of the wish to die, the desire to make an actual suicide attempt, and details of plans, if any; internal deterrents to an active attempt; and subjective feelings of control or “courage” regarding a proposed attempt. Each item consisted of three alternative statements graded in intensity from 0 to 2. Suicidal ideation was analysed dimensionally with scores ranging from 0 (low ideation) to 38 (high ideation) [6]. In other words, a positive rating (> 1) on any of the ideation scale’s 19 items was considered as a potential indicator of suicide ideation. Out of 29 items, 16 had positive and significant item-total correlations, and a Cronbach alpha of .89 was obtained, which indicated the high reliability of the SSI and also supported the validity of this scale [6]. The validity of SSI was also indicated by the moderate correlations with clinical ratings of suicidal risk and self-harm [7]. The scale was pilot tested and the result yielded a Cronbach’s alpha of .82.

#### Brief COPE (B-COPE [12])

The B-COPE provides researchers a way to quickly assess potentially important coping responses. It consists of 14 sub-scales, each of two items. Therefore, B-COPE has a total of 28 items, which measure 14 conceptually differentiable coping skills. Some of these skills are known to be generally adaptive (such as active coping, planning, positive reframing, acceptance, humor, religion, emotional support-seeking, and instrumental support-seeking); others are known to be problematic or maladaptive (such as self-distraction, denial, venting, substance use, behavioral disengagement, and self-blame). The response options ranged from 0 (I haven’t been doing this at all) to 3 (I have been doing this a lot). Researchers have variously shown B-COPE to have had good psychometric properties [24, 28]. All dimensions demonstrated good internal consistency (.70) in our pilot testing of the scale, with the exception of religion (=.63) and venting (=.61). We re-analyzed the adaptive and maladaptive dimensions, and they both showed high reliability (=.85 and.79, respectively). Higher score on the adaptive dimension indicates higher adaptive measures, while a lower or moderate score on the maladaptive dimension indicates adaptive coping.

#### Resilience scale (RS-14 [72])

RS-14 measures the capacity to withstand life stressors and derive meaning from them. It contains items which measure two major dimensions of psychological resilience: personal competence (as indicated in items 1, 2, 5, 6, 7, 8, 9, 11, 12, 14), and acceptance of self and life (as indicated in items 3, 4, 10, 13). It has a composite internal consistency reliability of .93. All the 14 items were positively worded, and participants responded on a 7-point scale that ranged from “strongly disagree” (1) to “strongly agree” (7). We obtained a Cronbach’s alpha of .90. A higher score on RS-14 indicates greater resilient capacity.

#### Statistical analysis

The research was a survey. A Pearson’s Correlation (*r*) analysis was conducted to examine the relationships between the demographic factors both with themselves and the other independent and dependent variables in the study. The reason for the choice of correlation is based on Urbina’s [65] assertion that correlations play a major role in demonstrating linkages between (a) scores on different tests, (b) test scores and non-test (demographic) variables, (c) scores on parts of tests and scores on whole tests, etc. [65]. Demographic variables such as gender were dummy coded before they were included in the correlation analysis. Dummy coding was recommended by experts in statistics as very important in correlation and regression as a “way of representing people using only zeros and ones” [23]. In order to clearly test the hypotheses, the study variables were submitted to a hierarchical regression analysis. Hierarchical regression analysis allows researchers to simultaneously examine the contributions of each of several predictor variables in one study. In the regression analysis, the demographic variable of marital status that was significantly correlated with suicidal ideation was first included in the analysis in order to control for its possible effect. This formed Step 1 in the analysis. Thereafter, academic stress was included in the regression to test for its predictive association with suicidal ideation, and this formed Step 2 of the analysis. Afterwards, the adaptive dimension of the coping strategy (which was considered as a separate entity) was included in the analysis, and this formed Step 3. Subsequently, the maladaptive dimension of the coping strategy was added to the analysis and this formed Step 4. Finally, resilience was added to the analysis and that formed Step 5. These variables were “entered” “step-by-step” (separately) into the analysis in order to examine the various respective accounts or percentage contributions of each predictor variable in the relationship [50].

The Hayes regression-based PROCESS macro was used to test for the moderation relationships. The PROCESS macro was chosen because it offers the opportunity to determine the interaction effect by generating a series of plots that can be later put together into a diagram or graph. The diagram further illustrates the conditional effect of X (main predictor) on Y (dependent variable), as a function of M (moderator variable). The moderating effects are thereafter examined using the regions of significance in accordance with the Johnson-Neyman technique. Process is a better choice for research where the variables are all directly measured (e.g., in clinical, health, and psychological settings that use hard data). All analyses were conducted using the Statistical Package for Social Sciences (SPSS) version 22 [42, 61].

## Results

From the results of the brief descriptive statistics (Table 1) performed on the demographics like age, marital status, and religion, the ages of the participants ranged from 18 to 32 years, with a mean age of 25 years and a standard deviation of .45. A total of 492 (97.4%) were single, while 7 (1.4%) were married. The number of Christians in the sample was 486 (96.2%), traditional 17 (3.4%), and Islam 2 (.4%). Age, marital status, religion, etc., that have been either positively or negatively implicated in suicidal ideation [1, 4], were included in the preliminary stage of the analysis. Inclusion criteria included full-time registered, non-working class undergraduate students of federal universities under study, while exclusion criteria included working class and postgraduate students who were known to be registered students of federal universities under study.

**Table 1:** Descriptive statistics of the participants

S/N	Variables	Frequency (f)	Percent (%)
1	Sex		
	Males	329	65.1
	Females	176	34.9
2	Age		
	18-22 yrs	383	75.8
	23-27 yrs	11.8	23.4
	28 yrs. and Above	4	8
3	Single		
	Marital Status	492	97.2
	Married	7	1.4
4	Ethnic Group		
	Igbo	459	90.9
	Hausa	8	1.6
	Yoruba	18	3.6
	Others	20	4.0
5	Religion		
	Traditional	17	3.4
	Christianity	486	96.2
	Islamic	2	.4

Results in Table 2 showed that suicidal ideation had a positive association with marital status (*r* = .08, p.05) but did not correlate with gender, age, or ethnic group. Academic stress (*r* = .17; p.001) was found to be positively related to suicidal ideation, whereas resilience (*r* = −.22; p.001) was found to be negatively related to suicidal ideation. Gender, age, and ethnic group that did not correlate with suicidal ideation were excluded from the analysis, and marital status, which correlated with suicidal ideation, was controlled in the subsequent moderation analysis. Suicidal ideation had no significant relationship with adaptive coping style (*r* = −.02), but it did have a significant relationship with maladaptive coping (*r* = .15; p.001).

**Table 2:** Mean, standard deviation and correlation results of academic stress, resilience and coping (Maldaptive and adaptive) on suicidal ideation

	(Mean, SD)	1	2	3	4	5	6	7	8	9
1. Suicidal Ideation	(5.86, 5.94)	1.00								
2. Sex		−.00	1.00							
3. Age		−.03	.01	1.00						
4. Marital Status		.08*	−.09*	−.07	1.00					
5. Ethnic Group		.05	−.14**	.05	.35***	1.00				
6. Academic Str.	(45.46, 12.39)	.17***	.15**	.01	−.10*	−.02	1.00			
7. Resilience	(75.28, 17.48)	−.22***	.16***	.04	−.09*	−.12**	−.11***	1.00		
8 ACS	(35.98, 5.90)	−.02	.08*	.05	.03	−.04	−.03	.11***	1.00	
9. MALCS	(34.34, 7.37)	.15***	−.02	.05	.00	−.08*	.32***	.02	.19***	1.00

*SD* standard deviation, *ACS* adaptive coping style, *MALCS* maladaptive coping style, *Str* stress

^*^p < .05

^**^p < .01

^***^p < .001

Table 3 indicates that subscales of coping were correlated with suicidal ideation. The separation of B-COPE into adaptive and maladaptive styles was done after the various dimensions of adaptive and maladaptive coping styles were correlated with suicidal ideation as shown in Table 3. Subsequently, strategies that negatively correlated (active, planning, positive refraining, acceptance, religion, and emotional support) with suicidal ideation were indicated as protective or adaptive and were summed together, while strategies that positively correlated (humour, instrumental support, self-distraction, denial, venting, behavioural disengagement, self-blame, and substance use) with suicidal ideation were maladaptive and summed together. This categorization is in line with extant literature (e.g. [5, 47]), which specifically suggested and categorized substance use (such as alcohol) as a maladaptive coping style because it impairs judgment and disinhibits impulses, and as such, users of such substances are more likely to harm themselves or die by suicide.

**Table 3:** Correlation results between B-COPE and suicidal ideation

S/N	Suicidal Ideation	Pearson r	Sig (2-tailed)
**1**	**Active**	−.04	.32
**2**	**Planning**	−.03	.47
**3**	**Positive refraining**	−.04	.38
**4**	**Acceptance**	−.07	.15
**5**	**Humour**	.02	.68
**6**	**Religion**	−.10*	.03
**7**	**Emotional support**	−.03	.46
**8**	**Instrumental support**	−02	.73
**9**	**Self-distraction**	−07	−12
**10**	**Denial**	.07	−10
**11**	**Venting**	.07	.12
**12**	**Substance**	.09**	**.02**
**13**	**Behavioural disengagement**	.18**	.00
**14**	**Self-blame**	.10*	.02

^*^p < .05

^**^p < .001

Results in Table 4 showed that Step 1, which involved only the demographic variable (marital status), revealed no significant result. R = 08, (R2 = .01), F(1, 503) = 2.93. Step 2 yielded a significant result: R = .19, (R^2^ = .04), F (1, 502) = 16.18, *p* = .01. The results showed that the addition of academic stress accounted for an additional 3 % of significant variance in suicidal ideation. ΔR2 = .03, F (1, 502) = 16.18, *p* = .01. However, Step 3 did not yield any additional significant results. Δ*R*^2^ = .04, (R^2^ = .06, F (1, 501) = .09. Step 4 produced a significant overall model, with R = .22, (R^2^ = .05), F (1, 500) = 5.44, *p* = .00. This means that the inclusion of a maladaptive coping style accounted for 1% of the significant variance in suicidal ideation, R2 = .01, F (1, 500) = 5.44, *p* = .00. And finally, Step 5 yielded a significant result, R = .29, (R^2^ = .08), F (1, 499) = 19.94, *p* = .00. Furthermore, the inclusion of resilience accounted for an additional 4% variance in suicidal ideation, R2 = .04, F (1, 499) = 19.94, *p* = .00.

**Table 4:** Hierarchical regression model results of academic stress, resilience and coping (Maldaptive and adaptive) on suicidal ideation

Predictor	B	Standard Error of B	β	t	***F***	R^**2**^
**Step I**						
Marital Status	1.23	.72	.08	1.71	2.93	.01
**Step 2**						
Academic Stress	.09	.02	.18	4.02***	16.18	.04
**Step 3**						
ACS	−.01	.04	−.01	−.29	.09	.04
**Step 4**						
MALCS	.09	.04	.11	2.33***	5.44	.05
**Step 5**						
Resilience	−.07	.02	−.20	−4.47***	19.94	.08

*ACS* adaptive coping style, *MALCS* maladaptive coping style, *B* standardized beta coefficient, *β* unstandardized beta coefficient, *t* total, *F* f-ratio, *R*^2^ R Squared

^***^p < .001

**Fig. 1:**
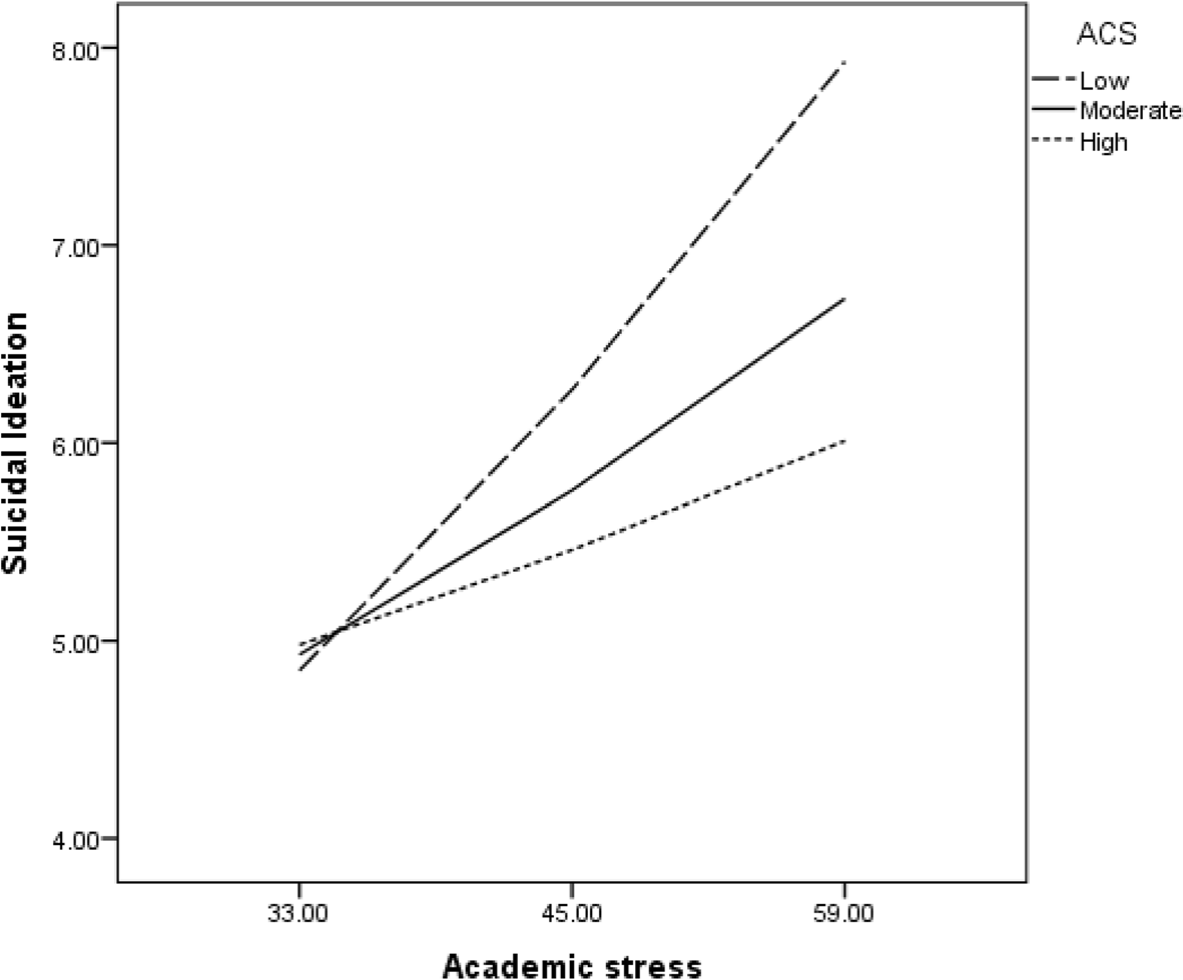
Adaptive coping style moderating the link between academic stress and suicidal ideation

**Fig. 2:**
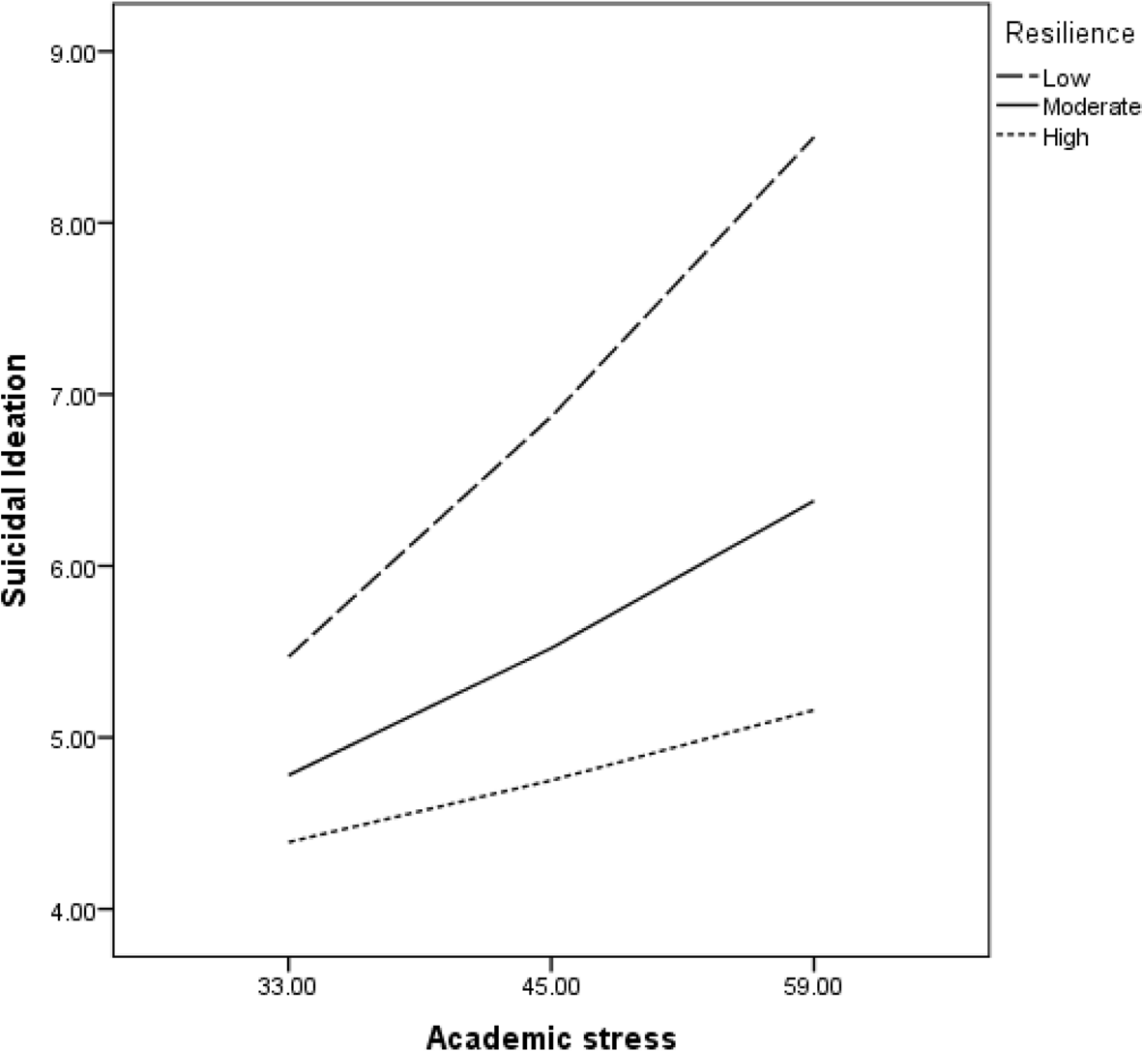
Resilience moderating the link between academic stress and suicidal ideation

Results in Table 4 indicated that marital status, which was initially correlated with suicidal ideation, failed to predict suicidal ideation. However, academic stress was a significant predictor of suicidal ideation. At low (B = .12, t = 4.40, *p* < .001) and moderate (B = .02, t = 3.19, *p* < .01) levels of adaptive coping style, academic stress was positively associated with suicidal ideation, but the association between academic stress and suicidal ideation was not significant at high levels of adaptive coping style (B = .03, t = 1.41, *p* < .16), (Fig. 1). Suicidal ideation, on the other hand, was negatively predicted by resilience (B = −.07, SE = .02, p.001). However, academic stress was positively associated with suicidal ideation at low (B = .12, t = 3.93, *p* < .001) and moderate (B = .06, t = 2.91, *p* < .01) levels of resilience; but for those students with high resilience, academic stress was not associated with suicidal ideation (B = .03, t = 1.08, *p* < .281), (Fig. 2). To avoid potentially problematic high multi-collinearity with the interaction terms, the variables were centered and an interaction term between adaptive coping style and suicidal ideation as well as the interaction between resilience and suicidal ideation were created [3]. Examination of the interaction plots is illustrated in Figs. 1 and 2.

## Discussion

Our hypothesis that academic stress would significantly predict suicidal ideation was confirmed, and this finding is consistent with extant studies [10, 18, 39]. Other literature (e.g [31, 48, 66]) have also documented the significant relationship between academic stress and suicidal ideation. In line with our goal of examining the moderating roles of coping styles in furthering research knowledge about suicide, intervention, and treatment, we found that adequate coping with academic stressors was key to avoiding suicidal ideation among students. This finding is very important as educational administrators and policy makers should incorporate courses and teachings of effective coping skills into their programs, especially for young students since stressors are inherent in the lives of undergraduate students, especially in our society and at this perilous time.

Stressors have become so prevalent in undergraduate education [19, 22, 49] that adequate coping skills have become a panacea to the likelihood of impending suicidal ideation [31, 48]. We found that adaptive coping styles did not significantly predict suicidal ideation, but moderated the relationship such that low or moderate coping with academic stress would most likely lead to suicidal ideation. Students are mostly confronted with the challenges of adapting to a new academic environment, academic workload, academic performance, attending to lectures, overwork, or thoughts of future employment after graduation [22, 49], and most seriously, social, emotional, and financial stress [19]. This is also in consonance with worries about unemployment rates, poverty and destitution, economic crises, feelings of insecurity, marginalization, and economic disempowerment [8, 44] that dominate our society today. It is made even worse when the student(s) loses their guardian/parents/sibling who pays their academic bills, or when the guardian/parents/sibling suffers a misfortune that renders him/her almost destitute.

Resilient students have the ability to recover from stress [71], but not without adequate coping strategies. Our study found that resilience was positively associated with academic stress and negatively predicted suicidal ideation. Thus, the hypothesis which stated that resilience would moderate the relationship between academic stress and suicidal ideation was confirmed. This simply means that those who cope well with academic stress have a better chance of bouncing back than those who do not, and they are less likely to consider suicide. In line with our findings, Tugade et al. [64] noted that resilient people have much more adaptive behaviours, particularly in the areas of social functioning, morale, and somatic health, and such people equally experience positive emotions amidst stress; given that moral and social functioning are anti-suicidal tonics. The resiliency theory proposed by Richardson [51] explains that qualities of resilience such as optimism, hopefulness, and meaningful engagement ensure higher immune levels than helplessness, hopelessness, and depression (which are precursors of suicide). Therefore, resilience promotes succor and adequate coping under threats of various academic stressors.

Our findings can be explained by Aaron Anthonovsky’s Salutogenic Model of Resilience. In its explanations of resilience, the salutogenic model ignores the whole notion of risk exposure as a prerequisite for being labelled “resilient” and instead places the emphasis on factors that contribute to health and wellbeing. The salutogenic model specifically focuses on factors that help identify coping resources that may contribute to resilience and effective adjustment, notwithstanding adversity and risk [60]. It is adequate coping skills that make resilient students able to quickly regain a sense of balance that keeps them going despite academic difficulty and trouble, and equally makes them find meaning amidst academic confusion and turmoil. Resilient students are self-confident and understand their own strengths and abilities. They do not feel a pressure to conform but take pleasure in being unique. Extant literature have documented the relationships between resilience and well-being [40]. Perceived resilience was associated with less suicidal ideation whereas higher psychological distress was associated with higher suicidal ideation [16], depression, anxiety, mental health, resiliency, and daily stresses had been linked to suicidal ideations and are noted to play significant role in suicidal ideation [32]. To our knowledge, it seems that no study had particularly evaluated the moderation of coping and resilience on the path of academic stress and suicidal ideation. Hence, our study becomes an interesting read for students, educational administrators, and some other non-governmental suicidal organizations.

Our study is not without limitations. For instance, the small sample size of our study may not have been large enough to account for generalizations across cultures. In the same vein, university differences in terms of courses offered, and socioeconomic status, which definitely would have ensured a more homogenous population, were not factored in the sampling process. Subsequent studies should consider such university differences and capture course types that might impact on academic stress and suicidality. Again, as a cross-sectional study, our data do not allow for full inferences about causal directionality. As a self-report measure was adopted in the study, there is the possibility of response biases as participants may either have made socially acceptable answers rather than being truthful or were unable to accurately assess themselves; all these threaten the reliability and validity of the measurement. Equally, tools employed in this study, like the Lakaev Academic Stress Response Scale, Scale for Suicidal Ideation, Brief COPE, and Resilience Scale, cannot be viewed as diagnostic tools, but only as screening tests to identify members of groups at risk for these conditions. The results arising from these tools tell us how the students perceive their health but are not in themselves evidence of medical concerns. Therefore, future studies should consider making more directional inferences, perhaps from a more controlled experimental investigation as well as cross cultural variances in suicidal ideation. We did not also take into account several ways people ideate about suicide (e.g., active ideation with plans, thoughts of suicide, and urges) as noted by Rizvi and Fitzpatrick [56]. There is a likelihood that the frequency, duration, intensity, and future possibility of these ways of ideating suicide could have been propagated by the academic environment and that most students at different times have the urges and thoughts (with or without) active plans. This area should be explored further. Finally, **s**election bias could undermine the internal validity of the study. However, the use of this approach might not have a significant impact on the outcome of this study. Nonetheless, this can only be ascertained when further studies are conducted while taking into consideration the issues raised. We acknowledge this as a limitation of the sampling technique adopted and advise the exercise of caution in making generalizations from these findings.

Based on the limitations stated above, it is recommended that future studies ensure adequate representativeness, increased homogeneity, etc. in order to foster generalizations of the findings.

## Conclusion

Resilient students having the ability to recover from stress are only possible with adequate coping strategies even as resilience positively associated with academic stress and negatively predicted suicidal ideation. Our findings affirm the research trend that academic stress is associated with suicidal ideation, with resilient students able to bounce back from academic challenges. Good coping strategies also enable resilient students recover from stress, consequently reducing their likelihood to ideate about suicide. Our students must adopt positive coping strategies towards solving their academic problems and learn persistence in the midst of threatening academic situations.

Our findings contribute to the growing evidence that adequate coping with academic stressors and resilient skills are keys to avoiding suicidal ideation among young students. Resilient students with adequate coping strategies find it easier to recover from stress even as resilience is positively associated with academic stress and negatively predicted suicidal ideation. This simply indicates that those who cope well with academic stress have more chances of bouncing back than others who do not, and may not likely ideate about suicide.

## Data Availability

The datasets generated and/or analyzed during the study are available from the corresponding author based on special request and the corresponding author should be contacted via this email: kalu.ogba@unn.edu.ng.

## References

[1] Adewuya AO, Oladipo EO. Prevalence and associated factors for suicidal behaviours (ideation, planning, and attempt) among high school adolescents in Lagos, Nigeria. Eur Child Adolesc Psychiatry. 2020:29(11):1503–12. 10.1007/s00787-019-01462-x 31858265

[2] Adeyemo S, Adeosun II, Ogun OC, Adewuya A, David AN, Adegbohun AA (2020). Depression and suicidality among adolescents living with human immunodeficiency virus in Lagos, Nigeria. Child Adolesc Psychiatry Ment Health.

[3] Aiken LS, West SG (1991). Multiple regression: testing and interpreting interactions.

[4] Akinremi R (2020). Nigeria has highest suicide rate in Africa, sixth globally.

[5] Bagge CL, Conner KR, Reed L, Dawkins M, Murray K (2015). Alcohol use to facilitate a suicide attempt: an event-based examination. J Stud Alcohol Drugs.

[6] Beck A, Kovacs M, Weissman A (1979). Assessment of suicidal intention: the scale for suicidal ideation. J Consult Clin Psychol.

[7] Beck A, Steer R, Renieri WF (1988). Scale for suicidal ideation: psychometric properties of a self-report version. J Clin Psychol.

[8] Best S (2003). A beginner’s guide to social theory.

[9] Bilsen J (2018). Suicide and youth: risk factors. Front Psychiatry.

[10] Breton JJ, Labelle R, Berthiaume C, Royer C, St-Georges M, Ricard D, Guilé JM (2015). Protective factors against depression and suicidal behaviour in adolescence. Can J Psychiatr.

[11] Busch KA, Fawcett J, Jacobs DG (2003). Clinical correlates of inpatient suicide. J Clin Psychiatry.

[12] Carver C (1997). You want to measure coping but your protocol’s too long: consider the brief- COPE. Int J Behav Med.

[13] Çevik H (2020). Investigating the relationship between perceived stress and leisure coping strategies among university students: Eskisehir Technical University case. Int Educ Stud.

[14] Chia BH. Too young to die: An Asian perspective on youth suicide paperback. Marshall Cavendish Intl. USA. 1999.

[15] Chou WP, Yen CF, Liu TL. Predicting Effects of Psychological Inflexibility/Experiential Avoidance and Stress Coping Strategies for Internet Addiction, Significant Depression, and Suicidality in College Students: A Prospective Study. Int J Environ Res Public Health. 2018;18;15(4):788. 10.3390/ijerph15040788 PMC5923830 29670025

[16] Cleverely K, Kidd SA (2011). Resilience and suicidality among homeless youth. J Adolesc.

[17] Clum GA, Febbraro GA (1994). Stress, social support, and problem-solving appraisal/skills: prediction of suicide severity within a college sample. J Psychopathol Behav Assess.

[18] Consoli A, Cohen D, Bodeau N, Guilé JM, Mirkovic B, Knafo A (2015). Risk and protective factors for suicidality at 6-month follow-up in adolescent inpatients who attempted suicide: an exploratory model. Can J Psychiatr.

[19] DeRosier ME, Frank E, Schwartz V, Leary KA (2013). The potential role of resilience education for preventing mental health problems for college students. Psychiatr Ann.

[20] Dixon WA, Heppner PP, Rudd MD. Problem-solving appraisal, hopelessness, and suicide ideation: Evidence for a mediational model. J Couns Psychol. 1991;41(1):91–8.

[21] Dixon WA, Rumford KG, Heppner PP, Lips BJ (1992). Use of different sources of stress to predict hopelessness and suicide ideation in a college population. J Couns Psychol.

[22] El Ansari W, Khalil K, Stock C. Symptoms and health complaints and their association with perceived stressors among students at nine Libyan universities. Int J Environ Res Public Health. 2014;25;11(12):12088–107. 10.3390/ijerph111212088 PMC4276602 25429678

[23] Field A. Discovering Statistics Using SPSS. 3rd Edition, Sage Publications Ltd., London. 2009.

[24] Fletcher K, Parker G, Manicavasagar V (2014). The role of psychological factors in bipolar disorder: prospective relationships between cognitive style, coping style and symptom expression. Acta Neuropsychiatr.

[25] Friedlander A, Nazem S, Fiske A, Nadorff MR, Smith MD (2012). Self-concealment and suicidal behaviors. Suicide Life Threat Behav.

[26] Freire C, Ferradás MDM, Valle A, Núñez JC, Vallejo G (2016). Profiles of psychological well-being and coping strategies among university students. Front Psychol.

[27] Glenn CR, Kleiman EM, Kellerman J, Pollak O, Cha CB, Esposito EC, Porter AC, Wyman PA, Boatman AE. Annual Research Review: A meta-analytic review of worldwide suicide rates in adolescents. J Child Psychol Psychiatry. 2020;61(3):294–308. 10.1111/jcpp.13106 31373003

[28] Greenhouse WJ, Meyer B, Johnson SL (2000). Coping and medication adherence in bipolar disorder. J Affect Disord.

[29] Hamzah M (2019). Re: Why have you used SPSS/Preacher & Hayes Macro instead of SEM? What could be the best response to this comment by a reviewer?.

[30] Horwitz AG, Hill RM, King CA (2011). Specific coping behaviors in relation to adolescent depression and suicidal ideation. J Adolesc.

[31] Howarth EJ, O'Connor DB, Panagioti M, Hodkinson A, Wilding S, Johnson J (2020). Are stressful life events prospectively associated with increased suicidal ideation and behaviour? A systematic review and meta-analysis. J Affect Disord.

[32] Izadinia N, Amiri, M.m Jahromi, G., & Hamidi, S. (2010). A study of relationship between suicidal ideas, depression, anxiety, resilience, daily stresses and mental health among Tehran university students. Proscenia Soc Behav Sci.

[33] Jackson D, Firtko A, Edenborough M (2007). Personal resilience as a strategy for surviving and thriving in the face of workplace adversity: a literature review. J Adv Nurs.

[34] Kalu TUO, Akande A, Adetoun B, Adewuyi M (2020). Socio-psychological implications of management and organizational bias and hate crime in Nigeria. The global nature of organizational science phenomena: sociocultural, environmental, and political context perspectives on international organizational behaviour issues.

[35] King RA, Schwab-Stone M, Flisher AJ, Greenwald S, Kramer RA, Goodman SH, Gould MS (2001). Psychosocial and risk behavior correlates of youth suicide attempts and suicidal ideation. J Am Acad Child Adolesc Psychiatry.

[36] Kyron MJ, Rikkers W, Page AC, O'Brien P, Bartlett J, LaMontagne A, Lawrence, D. Prevalence and predictors of suicidal thoughts and behaviours among Australian police and emergency services employees. Aust N Z J Psychiatry. 2021;55(2):180–95. 10.1177/0004867420937774 32615800

[37] Lakaev N. Validation of an Australian Academic Stress Questionnaire. Australian J Guidance Couns. 2006;19(1):56–70.

[38] Lambert VA, Lambert CE (2008). Nurses' workplace stressors and coping strategies. Indian J Palliat Care.

[39] Liang J, Kairi K, Bob L, Diego, De L, Lu Y, Mansor AT, Cun-xian J (2020). Coping strategies and suicidality: a cross-sectional study from China. Front Psychiatry.

[40] Mak W, Ng I, Wong C (2011). Resilience: enhancing well-being through the positive cognitive triad. J Couns Psychol.

[41] McKinnon B, Gariépy G, Sentenac M, Elgar FJ. Adolescent suicidal behaviours in 32 low- and middle-income countries. Bull World Health Organ.PubMed. 2016;94(5):340–50. 10.2471/BLT.15.163295 PMC4850530 27147764

[42] Mendenhall W, Beaver RJ, Beaver BM (2009). Introduction to probability and statistics.

[43] National Youth Policy of Nigeria- Revised 2009 (Federal Ministry Of Youth Development) Document of the Federal Republic of Nigeria 2009.

[44] Nweze A, Tanimu TN, Bashir IL, Alemiko EEO, Akano AO (1993). Psychological theories and policing. Policing Nigeria: past, present and future.

[45] O’Connor RC (2011). Towards an integrated motivational–volitional model of suicidal behaviour. Int Handbook Suicide Prev.

[46] Omigbodun O, Dogra N, Esan O, Adedokun B (2008). Prevalence and correlates of suicidal behaviour among adolescents in Southwest Nigeria. Int J Soc Psychiatry.

[47] Oxley J, Lenné M, Corben B (2006). The effect of alcohol impairment on road-crossing behaviour. Transport Res F: Traffic Psychol Behav.

[48] Panadero S, Martín R, Vázquez JJ (2018). Suicide attempts and stressful life events among homeless people in Madrid (Spain). J Community Appl Soc Psychol.

[49] Phang CK, Mukhtar F, Ibrahim N, Keng SL, Sidik SM (2015). Effects of a brief mindfulness-based intervention program for stress management among medical students:the mindful-gym randomized controlled study. Adv Health Sci Educ.

[50] Pompili M, Serafini G, Innamorati M, Dominici G, Ferracuti S, Kotzalidis GD, Serra G, Girardi P, Janiri L, Tatarelli R, Sher L, Lester D (2010). Suicidal behavior and alcohol abuse. Int J Environ Res Public Health.

[51] Richardson GE (2002). The meta-theory of resilience and resiliency. J Clin Psychol.

[52] Rossetti M, Tosone A, Stratta P, Collazzoni A, Santarelli V, Guadagni E, Rossi R, Rossi A (2017). Different roles of resilience in depressive patients with history of suicide attempt and no history of suicide attempt. Rev Bras Psiquiatr.

[53] Saadu UT (2019). An investigation into suicidal ideation of low academic achievers in Kwara State University, Malete, Nigeria. FUDMA J Educ Foundations.

[54] Shrout PE, Bolger N (2002). Mediation in experimental and nonexperimental studies: new procedures and recommendations. Psychol Methods.

[55] Swahn M, Bossarte R, Elimam DM (2010). Prevalence and correlates of suicidal ideation and physical fighting: a comparison between students in Botswana, Kenya, Uganda, Zambia and the USA. Int J Public Health.

[56] Rizvi SL, Fitzpatrick S. Changes in suicide and non-suicidal self-injuryideation and the moderating role of specific emotions over the course of dialectical behavior therapy. Suicide Life Threat Behav. 2020:1–17. 10.1111/sltb.12691. 10.1111/sltb.12691 32969037

[57] Skinner EA, Zimmer-Gembeck MJ. The development of coping. Annu Rev Psychol. 2007;58(1):119–44. 10.1146/annurev.psych.58.110405.085705 16903804

[58] Snowdon J, Choi NG. Undercounting of suicides: where suicide data lie hidden. Global Public Health. 2020:1–8. 10.1080/17441692.2020.1801789 32744898

[59] Speckens AE, Hawton K (2005). Social problem solving in adolescents with suicidal behavior: a systematic review. Suicide Life Threat Behav.

[60] Sun J, Stewart DE (2007). Development of population-based resilience measures in the primary school setting. Health Educ.

[61] Tabachnick BG, Fidell LS (2013). Using multivariate statistics.

[62] Tang F, Qin P (2015). Influence of personal social network and coping skills on risk for suicidal ideation in Chinese university students. PLoS One.

[63] Thompson R, Proctor LJ, English DJ, Dubowitz H, Narasimhan S, Everson MD. Suicidal ideation in adolescence: examining the role of recent adverse experiences. J Adolesc. 2011. 10.1016/j.adolescence.2011.03.003. 10.1016/j.adolescence.2011.03.003 PMC3743921 21481447

[64] Tugade M, Fredrickson B (2004). Resilient individuals use positive emotions to bounce back from negative emotional experiences. J Pers Soc Psychol.

[65] Urbina S (2014). Essentials of psychological testing.

[66] Uğurlu N, Ona N (2009). Relationship between the stress-coping levels of university students and their probability of committing suicide. Soc Behav Personal Int J.

[67] Van Heeringen K, vanHeeringen K (2001). The suicidal process and related concepts. Understanding suicidal behaviour.

[68] Yang B, Clum GA (1994). Life stress, social support, and problem-solving skills predictive of depressive symptoms, hopelessness, and suicide ideation in an Asian student population: a test of a model. Suicide Life Threat Behav.

[69] Yin Y, Tong J, Huang J (2020). Suicidal ideation, suicide attempts, and neurocognitive dysfunctions among patients with first-episode schizophrenia. Suicide Life Threat Behav.

[70] Waern M, Beskow J, Runeson B, Skoog I (1999). Suicidal feelings in the last year of life in elderly people who commit suicide. Lancet.

[71] Wagnild GM (2009). The resilience scale user’s guide for the US English version of the resilience scale and the 14-item resilience scale.

[72] Wagnild GM, Young HM. Development and psychometric evaluation of the Resilience Scale. J Nursing Meas. 1993;1(2):165–78. 7850498

[73] World Health Organization. Suicide in the world: global health estimates: World Health Organization; 2019. https://apps.who.int/iris/handle/10665/326948. License: CC BY-NC-SA 3.0 IGO

[74] World Health Organization. Suicide is complex. World Health Statistics 2020 visual summary. 2020. https://www.who.int/news-room/fact-sheets/detail/suicide.

[75] Zhang X, Wang H, Xia Y, Liu X, Jung E (2012). Stress, coping and suicide ideation in Chinese college students. J Adolesc.

[76] Zimmerman MA (2013). Resiliency theory: a strengths-based approach to research and practice for adolescent health. Health Educ Behav.

